# Media and Digital Technologies for Mixed Methods Research in Public Health Emergencies Such as COVID-19: Lessons Learned From Using Interactive Radio–SMS for Social Research in Somalia

**DOI:** 10.1177/1558689820986748

**Published:** 2021-01-21

**Authors:** Johanna Riha, Claudia Abreu Lopes, Naima Abdi Ibrahim, Sharath Srinivasan

**Affiliations:** 1 University of Cambridge, Cambridge, UK; 2 United Nations University, Kuala Lumpur, Malaysia; 3 Africa’s Voices Foundation, Nairobi, Kenya

**Keywords:** mixed methods, interactive radio, digital technology, evaluation, public health

## Abstract

Radio shows which invite audience participation via short message service (SMS)—interactive radio–SMS—can be designed as a mixed methods approach for applied social research during COVID-19 and other crises in low and middle income countries. In the aftermath of a cholera outbreak in Somalia, we illustrate how this method provides social insights that would have been missed if a purely qualitative or quantitative approach were used. We then examine the strengths and limitations associated with interactive radio–SMS through an evaluation using a multimethod comparison. Our research contributes an application of a mixed methods approach which addresses a specific challenge raised by COVID-19, namely utilizing media and digital technologies for social research in low and middle income countries.

Infectious diseases pose serious and complex threats to population health and well-being, spreading faster and possibly emerging more frequently than ever before (O’Dowd, 2007). The Coronavirus Disease 2019 (COVID-19) pandemic in addition to other recent outbreaks of international concern (e.g., Middle East Respiratory Syndrome, Severe Acute Respiratory Syndrome, the Zika, and Ebola virus diseases), have illustrated the importance of applied and time-sensitive social research which can directly inform, develop, and assess more contextualized biosocial outbreak response plans (Abramowitz et al., 2018; Gilmore et al., 2020; Johnson & Vindrola-Padros, 2017). COVID-19 now places unprecedented demand on novel social research methodologies or new applications of methodologies developed in other contexts to inform evidence-based public health responses. Social research methods need to be adapted to be deployed quickly in situations where access to respondents is restricted due to physical distancing measures and constraints on human movement.

Mixed methods research offers an important opportunity in the field of social research in public health emergencies. Integrating different types of data can contribute to a more holistic understanding of the social factors affecting disease transmission and response (Rohan et al., 2018). This article provides an example of a social research mixed methods approach which could be applied in other contexts to inform and help monitor the response to health crises or other emergencies in low- and middle-income countries (LMICs).

The COVID-19 response pillar of “risk communication and community engagement” refers to the multidirectional communication and engagement required through the preparedness, response, and recovery phases, in order to encourage informed decision making, positive behavior change, and the maintenance of trust (World Health Organization [WHO], 2020). To be effective, governments and agencies must tailor interventions carefully to local sociocultural realities (Palagyi et al., 2019). First, the modes and styles of communication employed must be suitable in local contexts, including LMIC factors like lower internet penetration, higher broadcast media consumption, lower literacy and languages/dialects in use. Second, health messaging content must be relevant to specific sociocultural beliefs and dominant active ideas in the community concerning COVID-19 (Dune et al., 2020). Finally, the delivery of messaging must also come from messengers that are trusted (Dada et al., 2019). Understanding community worldviews is essential for ensuring practices to minimize transmission, protect vulnerable groups, and support adequate patient care. Effective risk communication and community engagement interventions in LMICs therefore need to be informed by situated and timely social research (Questa et al., 2020).

In LMICs, two factors make social research more urgent and challenging. First, response strategies in these settings rely heavily on contextually tailored nonpharmaceutical interventions for mitigation and suppression—notably risk communication and community engagement—given relatively weak health systems (Walker et al., 2020). Second, the lack of rigorous health data and data systems infrastructure, and the uneven penetration and uptake of digital technologies despite the popularity of radio and social media, are challenges that require creative approaches to social research. The applied mixed methods approach introduced and evaluated in this article—interactive radio and short message service (interactive radio–SMS)—bears relevance in these contexts.

In this article, we illustrate the use of and evaluate the interactive radio–SMS method deployed in the wake of the 2017 cholera outbreak in Somalia. Initially, we provide an example of how interactive radio–SMS was used as a mixed methods approach to enrich social insights in the aftermath of a cholera outbreak in Somalia. We then evaluate the validity of the interactive radio–SMS method through a comparison with two other methods in the same setting (Shadish et al., 2002; Thyer, 2012). Our research contributes to the field of mixed methods by demonstrating how interactive radio–SMS as a mixed methods approach can be used for social research in the context of public health emergencies as well as other fields.

## Interactive Radio–SMS as a Mixed Methods Approach

Mass media combined with information and communication technologies presents an opportunity to rapidly gather a large volume of data on social beliefs and knowledge embedded in local contexts. In many societies, radio remains a dominant media channel (International Telecommunication Union, 2010; Winthrop & Smith, 2012). With the liberalization of mass media, radio broadcasts have gained new audiences as a result of more diversified languages, programs and types of operators (Martinez-Costa & Prata, 2017; Srinivasan & Lopes, 2016). At the same time, mobile phone penetration has risen sharply. Nearly, two thirds of the world’s population subscribe to mobile services (GSM Association, 2018), with skews toward wealthier, male and urban populations (Haenssgen, 2018). The deployment of mobile surveys in health emergency scenarios represents an opportunity to open a channel of communication with affected populations allowing quick and timely feedback.

Capitalizing on the increasing uptake of information and communication technologies, interactive radio–SMS offers an innovative method for gathering data from communities that are hard-to-reach by other means during public health emergencies. In these situations where rapid insights are needed, there should be a compromise between celerity of information and quality of qualitative data. SMS ensures the former at the expense of the latter due to the limited number of questions and short length of messages (Berman et al., 2017; Kaplan, 2006). Expanding the interactive radio data to include an SMS-based survey, collecting self-reported demographic and behavioral data, would allow for integration of qualitative data with quantitative data. This could potentially yield a richer understanding of sociocultural determinants of a range of behaviors, cultural practices, and social norms, than if singular mono-methods were used. Furthermore, this method could have important applications for social research in other fields beyond public health (e.g., education, livelihoods, gender equity, conservation).

We define the interactive radio–SMS as a mixed methods approach that involves the following steps: (1) gathering qualitative (e.g., knowledge, opinions) and quantitative data (e.g., self-reported demographic and behavior data) from radio audiences via SMS, (2) thematically analyzing these qualitative data and then transforming them into quantitative data, and which are then used to (3) explore statistical associations between emergent themes and demographic and other variables.

## Application of the Interactive Radio–SMS Method

### Study Setting

In 2017, the humanitarian crisis in Somalia deteriorated due to inadequate rainfall over several seasons. Somalia experienced one of the worst cholera outbreaks, with over 78,780 cases and over 1,150 deaths mainly among children younger than 5 years (WHO, 2017). Despite public health interventions and a national vaccination drive, cholera remains a recurring and major risk to vulnerable communities across Somalia. In this context, in 2018, we undertook a research project to apply and evaluate the interactive radio–SMS method as a social research tool for use in public health emergencies. The project involved understanding community perceptions of cholera risk and preparedness across communities in four high risk areas in Somalia (Kismayo, Beledweyne, Banaadir [Mogadishu], and Baidoa).

### Data Collection Tool

**Figure 1.**
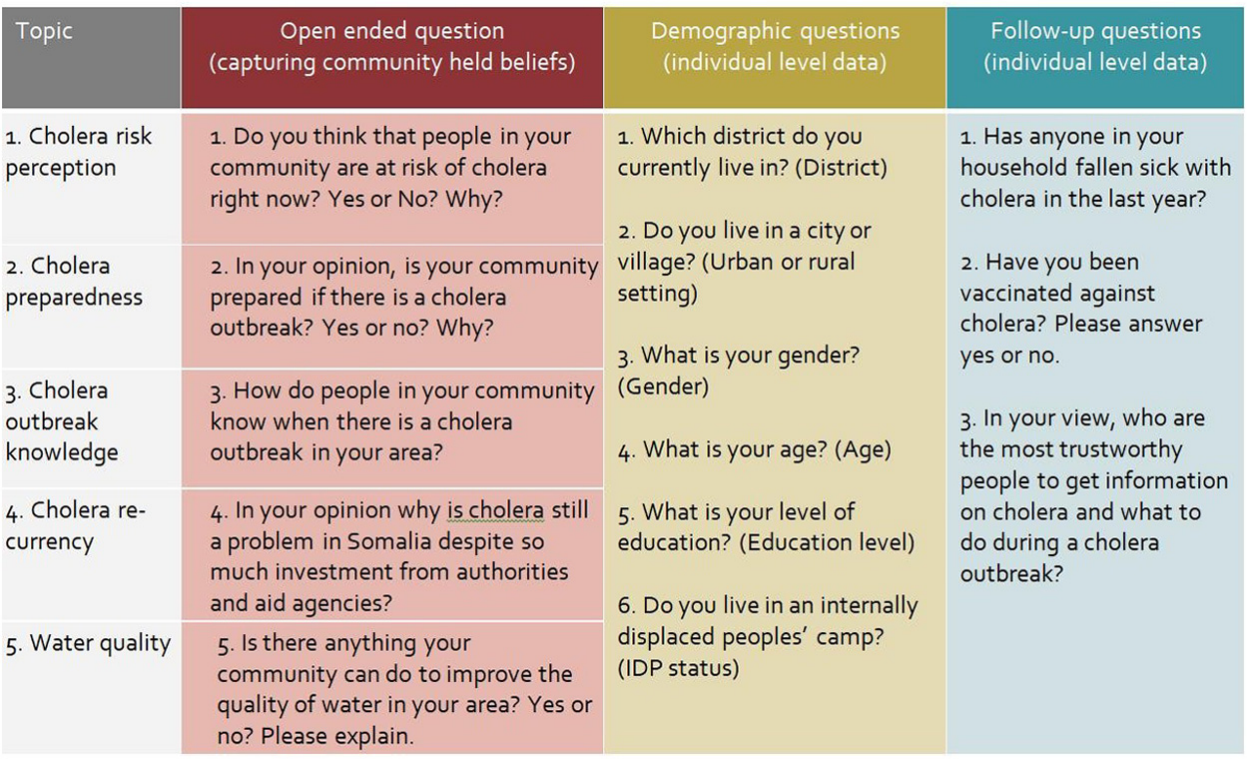
Outline of questionnaire used as part of the Cholera Outbreak Study.

Qualitative and quantitative data for this study were collected from SMS responses texted in by radio audiences. Figure 1 summarizes the questionnaire used in this study. Five open-ended questions aimed to capture community perceptions and beliefs of cholera risk, preparedness, knowledge of an outbreak, cholera recurrency, and water quality. Three follow-up questions were asked to capture occurrence of cholera cases within households, cholera vaccination exposure, and trustworthy sources of information during a cholera outbreak. Finally, demographic data were collected, which included age, gender, level of education, district of residence, residence in an urban or rural setting, and whether participants lived in an internally displaced people’s camp. All questions were pretested in Somali to assess people’s understanding of the questions in terms of cultural adequacy and language (accessibility and jargon-free), and making sure that questions were not open to misinterpretations.

### Data Collection

Between July and August 2018, 10 radio stations aired prerecorded 30-minute shows every Friday for 5 weeks with content themed around a weekly topic. Each week radio audiences were invited to text in their views on a different open-ended question corresponding to the weekly topic. To further boost participation from diverse demographic groups, for example, minorities or people whose voices are not usually heard on the radio, it was important to include audience views on the weekly topic in the prerecorded shows. To achieve this, all radio stations also aired short public service announcements three times a day every Sunday, Monday, and Tuesday prior to the Friday show, asking listeners to text in their views to the open-ended question of the week (to a free SMS shortcode), and to remind listeners of the upcoming show. In addition, weekly SMS advertisements with the open-ended question of the week and details of the upcoming show were sent to previous respondents prior to the upcoming Friday show. Selected audiences’ answers from these sources were then read out in the weekly pre-recorded radio shows.

**Figure 2.**
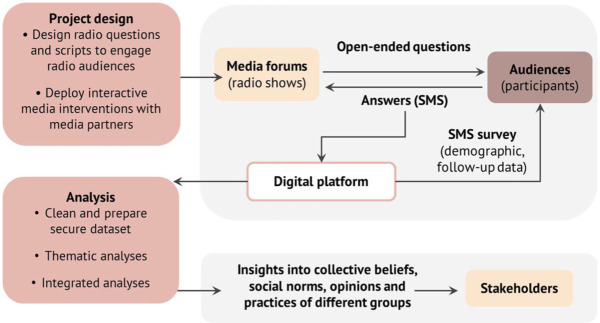
Schematic of how data are collected using the interactive radio–short message service method.

Once listeners texted in their response to an open-ended question, a cascade of one-to-one SMS questions was triggered to collect consent and quantitative demographic and follow-up information. All SMS interactions were free for listeners. Furthermore, all SMS responses were securely gathered and stored for analysis under a strict framework of data protection. Figure 2 provides a summary of the process of interactive radio–SMS data collection.

### Data Analyses and Integration

Since all data received through the interactive radio–SMS method were free-form SMS messages, raw data underwent preprocessing to structure the data set and to remove nonrelevant and repeat messages. The first step of preprocessing involved running all in-coming messages through an automated pipeline that created a comma separated file with prespecified variables and corresponding raw responses. Next, the file was processed through a data script to clean these data, creating new variables for these cleaned data. Repeat messages and nonrelevant messages (e.g., single letter or empty fields) were set to missing. For the quantitative variables, the data script automatically extracted words and attributed them numeric labels (e.g., “dheddig” [female in Somali] was coded as ‘1’) using prespecified regular expressions. The study team then reviewed and manually labeled messages that were not automatically coded.

For the qualitative responses, two Somali-speaking research assistants independently undertook a thematic analysis for each open-ended question. This was to minimize researcher bias during the thematic analysis. The Somali researchers were critical as they could interpret nuances and colloquial meanings. First, each researcher immersed themselves in the qualitative data by reading and rereading all responses to a specific open-ended question and taking notes. Next, they each generated initial descriptive codes to summarize respondents’ answers. For example, “no health services” and “no free medical centres” were codes developed to summarize reasons respondents gave for why they believed their communities were at risk of cholera. Each researcher then created subthemes and themes by identifying broad topics that codes clustered around. These lists of subthemes and major themes independently developed by the researchers were jointly reviewed and compared. Any discrepancies between them were discussed to reach a consensus and a final major-theme and subtheme list was developed. Specific verbatim quotes from respondents were then selected for each set of themes and subthemes to provide examples of the responses received and to reintegrate the voices of the respondents in the results.

To transform these qualitative data into quantitative data, a coding frame was constructed around these subthemes and themes. Next, the coding frame was applied independently by the two research assistants to 100 messages and the intercoder reliability was assessed by calculating Cohen’s kappa. For each open-ended question, the kappa value was above 0.80 indicating almost perfect agreement. Subsequently, all remaining messages were labelled using the relevant coding frame by a single Somali-speaking researcher using an in-house software interface. Following this labelling, each message was then assigned a series of binary codes for each major-theme and subtheme (“0” if the theme was absent and “1” if the theme was present), thereby converting the qualitative data into quantitative binary variables for subsequent analysis. Since themes were coded as discrete variables, logistic regression models were used to estimate associations (odds ratios) with demographic variables such as gender, age, and location, controlling for each other. Statistical control was used to avoid spurious associations related to different compositions of certain groups—for example, differing proportions of male and female participants in rural and urban areas.

These quantitative analyses were complemented with further qualitative interrogation, with insights illustrated by a selection of translated text messages. Quantitative analyses were completed using STATA Version 13.0 and R Studio Version 1.1.4.

### Results

Between July 2018 and September 2018, we received a total of 12,005 messages from 6,688 people who consented to take part in the project. Not all participants responded to all the open-ended or closed-ended questions, therefore sample sizes varied widely across questions (see Table 1). Of the respondents who replied to the relevant question, the majority were male, residents in urban areas, and youth (younger than 25 years of age). A little over 23% of 2,078 respondents reported living in an internally displaced people’s camp.

**Table 1.** The Number of People Who Provided Relevant Replies to Each Question and the Relevant Characteristic for Each Variable, Complete Interactive Radio–Short Message Service Data Set.

Variable	Number of people who provided a relevant response	Characteristic
Open-Ended Question 1	3,315	—
Open-Ended Question 2	1,201	—
Open-Ended Question 3	748	—
Open-Ended Question 4	1,111	—
Open-Ended Question 5	2,458	—
District	3,592	32.5% From Banaadir
Urban/rural	4,149	79.3% Urban residents
Gender	3,763	55.5% Male
Age	1,965	69.2% Have secondary or higher education
Level of education	1,677	69.6% Younger than 25 years of age
Living in IDP camp	2,078	23.3% Living in IDP camp
Household sickness	2,803	29.3% Someone in household sick with cholera in the last year
Cholera vaccination	2,677	28.7% Vaccinated against cholera

*Note*. IDP = internally displaced people.

To illustrate the types of social insights gained by using the interactive radio–SMS mixed methods approach, we present below findings mainly from the first open-ended question about cholera risk perception.

#### Qualitative Results

Of the 2,146 people who texted in their views in the first week, the majority reported that they felt their communities were at risk of cholera. Here are some examples of the audience views (translated) in response to the question about whether and why they think people in their community are at risk of cholera:

“Yes. There is no free access to free medical care facilities”

“Yes, because it is the hot season”

“Yes, because the water is dirty”

“Yes, because our people don’t take good care of sewages and dirt”

“Yes. People especially those living far from towns lack awareness”

“No, because we are people who are connected to their God”

“No, because the health workers have done great job in creating awareness”

“No, because the hot season is over”

“No, because most people have understood the causes of the disease and so it seems it is dying out”

“No. People in the neighbourhood have access to clean water and there is no garbage which is lying around”

“No, because we have good doctors and enough awareness”

**Figure 3.**
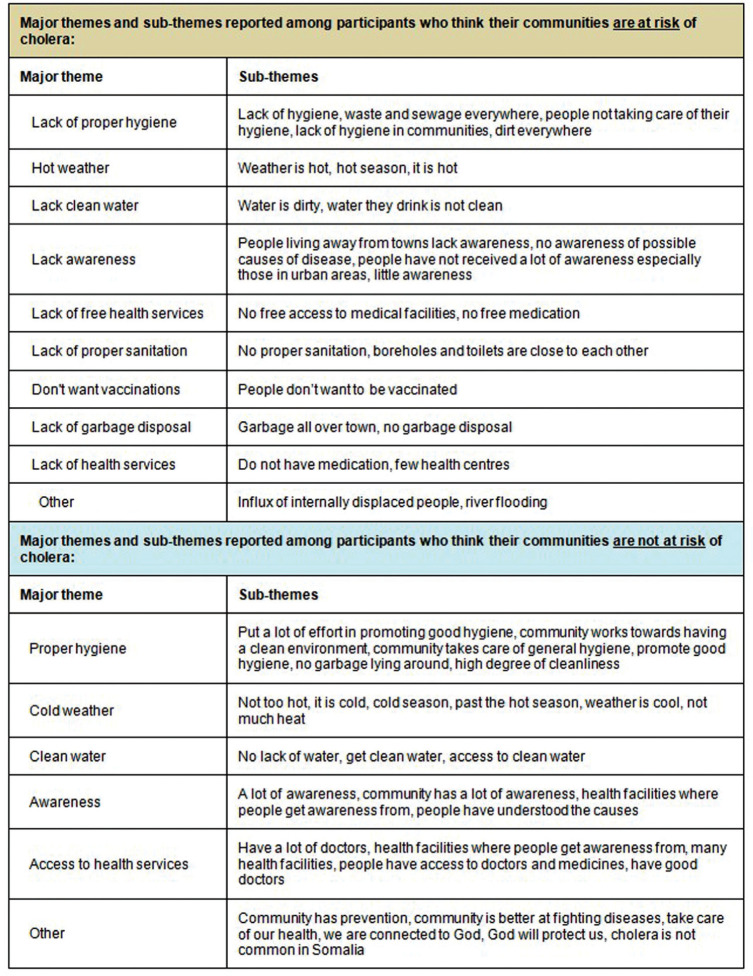
Summary of major themes and subthemes reported among participants who responded to the Week 1 open-ended question via the interactive radio–short message service method.

Figure 3 provides a summary of the major themes and subthemes reported as to why people did or did perceive their communities to be at risk of cholera.

Importantly, hygiene or lack of hygiene, was the most recurring theme among respondents who perceived their communities to be safe from cholera as well as those who perceived their communities to be at risk. Here are some of the respondents’ views:

“No, we practice good hygiene in the neighbourhood”

“No, because we put a lot of effort into hygiene, we wash the toilets with chlorine and we treat our water with chlorine”

“Yes, because there is waste and sewage everywhere”

“Yes, because there are poor hygienic condition in the camps”

“Yes, because people are not taking care of their hygiene”

This is really important from a public health perspective since good hygiene is critical for cholera prevention and control.

Interestingly, weather came up as an important theme among respondents, with respondents reporting cold weather being associated with less cholera in communities while hot weather was associated with cholera outbreaks. Here are some examples of what respondents said:

“No, because we are in the cold season and mostly cholera outbreaks happen during the hot season”

“We don’t expect cholera this year as it is not hot”

“Yes, it is the hot season now and people are using dirty water”

#### Integrated Results

Qualitative findings were further enriched when qualitative data were transformed and integrated with demographic data. For example, we found that

- The odds of reporting that their communities were at risk of cholera were 1.67 times higher for respondents from rural areas compared with those from urban areas (odds ratio = 1.67, 95% confidence interval [1.00, 2.79], *p* = .050, controlling for age, gender, location [classified by city], and internally displaced person’s status).
- The odds of reporting lack of clean water as a reason why their communities were at risk of cholera were 6.23 times higher for rural residents compared to urban residents (odds ratio = 6.23, 95% confidence interval [1.64, 23.61], *p* = .007, controlling for age, gender, location [classified by city], and internally displaced person’s status).
- The odds of reporting that their communities were at risk of cholera were 0.33 times lower for respondents who believed risk of cholera was dependent on the weather compared with those who did not mention weather (odds ratio = 0.33, 95% confidence interval [0.13, 0.83], *p* = .019, controlling for age, gender, location [classified by city], where people live [rural or urban], and internally displaced person’s status).

### The Need for an Evaluation of the Interactive Radio–SMS Mixed Methods Approach

Findings from the integrated analyses provide further insights into additional dimensions of beliefs about disease risk, signaling interesting divisions for further exploration which would not have been identified through the sole use of a qualitative approach. These findings provide a strong justification for the application of a mixed methods approach. Other strengths of the method included (1) deployment utilizing existing technological infrastructure and media channels; (2) remote implementation and potential to reach populations who may otherwise be hard to reach (geographically or linguistically); (3) iterative and evolving discussions with communities, building on previous engagement (a potentially responsive method); and (4) depth and breadth of data, as it provides beliefs, knowledge, and opinions from a large group of people.

At the same time, our research demonstrated clear limitations of the interactive radio–SMS mixed methods approach. These included sampling biases due to self-selection of participants; coverage biases due to exclusion of individuals who do not have access/listen to the radio; and exclusion of those who do not have access to a mobile phone and those who are illiterate or cannot text. Furthermore, as documented in other research, response quality and completion rates are often a problem when mobile phone text messages are used to gather data (Berman et al., 2017; Kongsgard et al., 2014).

Without a robust evaluation of the interactive radio–SMS method, it is difficult to determine whether and in which situations the strengths and potential added value of the method are outweighed by the limitations. We therefore undertook an evaluation, in parallel, to assess the validity of interactive radio–SMS as a mixed methods approach through a multimethod comparison in the context of the original study in Somalia.

## Evaluating Interactive Radio–SMS as a Mixed Methods Approach

To assess the validity of the interactive radio–SMS method, we conducted an evaluation comparing its results with those from computer assisted telephone interviews (CATI) and focus group discussions (FGDs). For our multimethod cross validation, as we could not randomize individuals to a particular method, we used a quasi-experimental noncomparable groups design (Shadish et al., 2002) whereby the same questions were tested across all the three methods. In the following sections, we outline the evaluation methodology and the results.

### Selection of Methods

In order to select which two methods we would use to compare with the interactive ratio–SMS approach, we reviewed five possibilities (outlined in Table 2) based on the following criteria: social context (interpersonal, group setting, or public), anonymity (perceived anonymity or not), mode (text, voice, and face-to-face), literacy (academic or technology literacy), use of information communication technologies (phones) and sampling strategies.

**Table 2.** Summary of Possible Methods and Criteria for Cross-Validation of Interactive Radio–Short Message Service.

Method	Social context	Mode	Anonymity	Literacy	Technology	Sampling
IR-SMS	Public	Text	Yes	High	Yes	Self-selection (nonprobabilistic)
Radio and IVR	Public	Voice	Yes	Medium	Yes	Self-selection (nonprobabilistic)
SMS survey	Interpersonal	Text	Yes	High	Yes	Purposive (nonprobabilistic)
CATI	Interpersonal	Voice	Yes	Medium	Yes	Cluster (probabilistic)
Household surveys	Interpersonal	Face-to-face	No	Low	No	Stratified random (probabilistic)
FGDs	Small group	Face-to-face	No	Low	No	Convenience (nonprobabilistic)

*Note*: IR-SMS = interactive radio–short message service; IVR = interactive voice recognition; SMS = short message service; CATI = computer-assisted telephone interviews; FGDs = focus group discussions.

FGDs were selected to compare with the interactive radio–SMS method because of the overlap across a number of dimensions—both use nonprobabilistic sampling with respondents providing answers in a social context and discussing ideas with others perceived to be “like them.” The interactive radio–SMS method might be conceptualized as a large scale FGD, reaching more people using radio and information communication technologies.

As a second method of comparison with the interactive radio–SMS method, CATI surveys were selected as preferable. Somalia has one of the lowest rates of internet penetration in the world as only 2% of individuals used the internet in 2017 (International Telecommunication Union, 2017). Therefore, online methods such as online surveys were not appropriate. As mobile phone penetration is considerably widespread (48.8% in 2017, according to International Telecommunication Union [2017]), mainly through basic phones, an SMS survey or CATI were considered feasible. Since an SMS survey implies texting, it excludes segments of the population with low literacy (Chib et al., 2012). To assess the effects of the interactive radio–SMS method’s prerequisite for literacy, we chose to use voice as a comparator and therefore CATI surveys as a third method for comparison. Large scale face-to-face household surveys or semistructured interviews were also considered, but they were too costly to implement given security risks in the study setting.

### Study Setting

This evaluation is nested within the larger research project described above. Due to financial and security constraints, a single region in Somalia was selected for this evaluation. Banaadir region, which is coterminous with the capital city Mogadishu, was selected because of the anticipated volume of participation in radio shows using the interactive radio–SMS method. Furthermore, being an urban/peri-urban area, Banaadir presents fewer logistical challenges in recruiting and conducting FGDs and CATI.

### Summary of Data Collection

The same questionnaire used in the main study (outlined in Figure 1) was used across all three methods (FGDs, interactive radio–SMS, and CATI) to gather both qualitative and quantitative data. Below is an overview of how data were collected through the FGDs and CATI.

#### Focus Group Discussions

In this study, a total of six FGDs were completed—three male and three female groups. Participants for one male and one female FGD were recruited from internally displaced people’s camps in Banaadir, since they are disproportionately affected by cholera due to poor access to basic water and sanitation facilities. Using snowball sampling, individuals who were at least 18 years old and had never participated in the interactive radio–SMS shows were invited to take part in these two FGDs. In addition, two male groups and one female group were randomly selected from a list of interactive radio–SMS participants who consented to be contacted. We could not recruit enough women who participated in interactive radio–SMS shows to form a second FGD as many women declined. We therefore recruited an additional group of women from the general Banaadir population using targeted snowballing. All FGD participants gave their consent to take part in the FGD. Each FGD lasted between 60 and 90 minutes, and had between 8 and 10 participants in addition to two researchers, a moderator and a notetaker. Where possible, the moderator and notetaker were of the same gender as the participants. The topic guide was designed to mirror the questionnaire used through the interactive radio–SMS method.

Detailed Somali transcripts and audio recordings were taken from each FGD. These transcripts were then translated verbatim into English by a researcher fluent in both Somali and English and independently checked for accuracy by a second bilingual researcher. The authors read through all the transcripts and completed a thematic analysis for each open-ended question.

#### Computer-Assisted Telephone Interviews

Multistage sampling was used to randomly recruit a total of 1,330 adults in Banaadir. The population in Banaadir was stratified by host communities (target 1,160 adults) and residents in internally displaced people’s camps (target 160 adults). This sample size was calculated assuming overall we wanted a margin of error of no more than 3% for a 95% confidence interval.

For recruitment of those in host communities, potential participants were selected using a stratified random sampling of households in the 17 districts in Banaadir, proportional to the district size, with the primary sampling unit being the household. A household was defined as one or more people who live in the same dwelling/structure who share meals. Based on previous studies using CATI methods (Lau et al., 2018), we anticipated a minimum 40% response rate among people we contacted via telephone. We therefore needed to contact at least 1,856 adults overall. To select households, first a location near the district center was chosen. Then three random directions were selected. Using a systematic random method, every third house was selected along each direction. Iterations were repeated until the district boundary was reached or the required sample size was achieved. Where the district boundary was reached before the required sample size was achieved, the next line was selected and the process repeated.

Within each selected household, the enumerator randomly selected one adult (aged 18 years and older) based on the Kish grid method and invited him/her to participate in the study. To guarantee the proportion between men and women, the procedure alternated between genders in consecutive households. Any dwellings where no one responded were revisited on at least two more occasions to attempt recruitment before being recorded as no contact.

To enroll 160 internally displaced people, we randomly selected three internally displaced people’s settlements based on the 486 settlements identified by the Internal Displacement Profiling in Mogadishu Report April 2016 (United Nations High Commissioner for Refugees, 2016). At least 53 adults were enrolled from each of the three camps following the same multistage process for the host communities. Given the limited sample size, the three settlements were not representative of the entire population of internally displaced people in Banaadir and no claims will be made about the internal displaced population.

Once the phone numbers were collected from those recruited and informed consent obtained, trained Somali staff called participants on a preset day and time and reconsented participants before beginning the CATI. Since participants were invited to answer all open-ended questions sequentially (unlike interactive radio–SMS participants who are likely to only answer one or two), we minimized the biases associated with ordered effects in our CATI sample through a partial counterbalancing design using Latin Squares, with each question asked once in each position (1-5). This design does not deal with transfer effects (effects of being asked a question after a particular sequence of previous questions); however, we do not anticipate transfer effects to be significant as the gains in knowledge are similar across all questions. At the end of the interview, participants were read out the same information regarding cholera prevention and management as per the radio shows to ensure equal gains of information across methods. All responses were entered in a predefined and self-validating questionnaire on computers and, where consent was obtained, an audio recording of the interview was also stored. Each interview lasted around 30 to 45 minutes.

An identical process as outlined for the interactive radio–SMS method was used to code answers and undertake thematic analysis in CATI. Furthermore, identical steps of data cleaning, preprocessing, and integrated analyses were completed using Python, R Studio Version 1.1.4, and STATA Version 13.0.

### Multimethod Cross-Validation Analyses

#### Qualitative Data

An important threat to the credibility of data obtained from the interactive radio–SMS method is social desirability. As an interactive radio show is like a public forum, people may want to present themselves in ways that enhance their image. People may also lie due to concerns related to privacy or disclosure. Other possible threats to construct validity are mono-operation bias as one construct is operationalized by a single empirical referent and effects linked to the introduction of an innovation can breed excitement, energy, and enthusiasm that contribute to higher response rates (Shadish et al., 2002). To evaluate the credibility of interactive radio–SMS data, theme diversity for each of the open-ended questions was compared across the three methods. Unexpected answers and levels of consensus were explored among participants in the FGDs, CATI, and interactive radio–SMS. Quotes were used in some cases to demonstrate how findings and interpretations were arrived at through these data, as well as to bring the voices of participants directly into analyses.

#### Quantitative and Integrated Data

For the quantitative and integrated components of the interactive radio–SMS mixed methods approach, we focused on identifying threats to the well-established criteria of internal and external validity (Groves et al., 2014; Shadish et al., 2002). Multiple steps were used to assess the validity of the quantitative component of the interactive radio–SMS sample. First, completeness of demographic and follow-up variables within each data set (interactive radio–SMS and CATI) was compared. Second, to test the external validity of the interactive radio–SMS sample, demographic characteristics were compared with those from the CATI sample, calculating percentages within each demographic group. For CATI samples, 95% confidence intervals for percentages were calculated; this was used to reject the hypothesis that the proportions of CATI and interactive radio–SMS are equal if the interactive radio–SMS percentages did not fall within the CATI confidence interval range, while incurring an error of less than 5%. Third, we compared the percentages (level-oriented comparisons) for binary open-ended questions from interactive radio–SMS with the 95% confidence intervals of percentages for the same binary questions from CATI. Fourth, comparisons between associations (odds ratios) between open-ended questions and demographic characteristics across methods (structure-oriented comparisons) were undertaken, considering whether odds ratios from the interactive radio–SMS sample fell within the confidence interval for CATI. Next, binary logistic regression models were run to obtain associations between answers to open-ended questions and follow-up questions controlling for all demographic variables. Finally, we carried out a series of binary logistic regressions on a combined interactive radio–SMS and CATI data set to test whether the mode (CATI vs. interactive radio–SMS) had any impact on the responses, also controlling for all demographic variables.

### Results of the Evaluation

We begin with a summary of our findings, with subsequent sections providing more detailed results. Overall, qualitative analyses revealed very similar major themes across all three methods (FGDs, interactive radio–SMS, CATI). Unsurprisingly, qualitative data were richest in FGDs, followed by CATI, and then interactive radio–SMS, suggesting that face-to-face group discussions provide richer data than text or telephone conversations. We found more divergent views among interactive radio–SMS and CATI participants, compared with FGD participants, for responses to binary questions. Quantitative and mixed analyses underscore the low external validity of interactive radio–SMS. Our evaluation found a bias in interactive radio–SMS participation toward individuals that reported a cholera case within their household, suggesting that interactive radio–SMS could be used to oversample those affected by an outbreak. Mode did not affect the binary (yes or no) responses to the open-ended questions, further suggesting similarity across methods. There was however limited capacity for more detailed associational analyses of thematic data with demographic and follow-up data in the interactive radio–SMS sample due to small sample sizes.

### Qualitative Results

#### Diversity and Depth of Views

Across all methods (FGDs, interactive radio–SMS, and FGDs) very similar major themes emerged in responses to the open-ended questions. Table 3 summarizes these main themes. For example, among FGDs, interactive radio–SMS, and CATI participants who reported that their communities were not prepared for a cholera outbreak, lack of awareness, lack of health services, and lack of clean water were major reasons why they felt their communities were not prepared. Furthermore, across all methods, poverty, and inability to afford treatment and prevention were intertwined with some of the other reasons why people believed communities could not be prepared:

“No they don’t have the things to prevent the disease and can’t afford.” (Interactive radio–SMS participant)

“No because mostly the poor have no access to hospital facilities.” (CATI participant)

“No, because mostly the people that it affects are the poor or the ones who live in the camps and the children are left alone at home all day and they can get cholera very easily.” (FGD participant)

**Table 3.** Major Themes Reported by Participants in Response to the Four Open-Ended Questions for Computer-Assisted Telephone Interviews, Focus Group Discussions and Interactive Radio–Short Message Service Data Sets.

		CATI	FGDs	IR-SMS
Open-Ended Question 1	Yes	• Lack of proper hygiene • Hot weather	• Lack of proper hygiene and sanitation • Hot weather	• Lack of proper hygiene • Lack of clean water
	No	• Proper hygiene	• Awareness • Proper hygiene	• Proper hygiene
Open-Ended Question 2	Yes	• Good hygiene • Awareness • Access to health services	• Good hygiene • Awareness • Access to health services	• Good hygiene • Community collaboration
	No	• Poor hygiene • Lack of awareness	• Poor hygiene • Lack of awareness	• Poor hygiene • Lack of awareness
Open-Ended Question 3	—	• Symptoms in people • Radio stations • People in health centers • Word of mouth	• Symptoms in people • Radio stations • People in health centers • Neighbors	• Symptoms in people • Affects children
Open-Ended Question 4	—	• Poor hygiene • Lack of awareness	• Poor hygiene • Lack of awareness	• Poor hygiene • Lack of awareness

*Note*. CATI = computer-assisted telephone interviews; FGDs = focus group discussions; IR-SMS = interactive radio–short message service method.

For many of the responses to the open-ended questions, more diverse themes were reported by participants in FGDs, followed by CATI and finally interactive radio–SMS (Table 4). For example, themes that came up in FGDs and/or CATI responses to Open-Ended Question 1 that did not appear in interactive radio–SMS included factors related to nutrition and cleanliness of food (both CATI and FGDs), overpopulation (both CATI and FGDs), children playing with mud (FGDs only), worms and flies spreading disease (FGDs only), and lack of a stable government (CATI only).

**Table 4.** Theme Diversity for Computer-Assisted Telephone Interviews, Focus Group Discussions, and Interactive Radio–Short Message Service Data Sets.

		CATI	FGDs	IR-SMS
Open-Ended Question 1	Yes	14 Themes	15 Themes, 9 common with CATI	7 Themes, no unique themes
	No	10 Themes	6 Themes, 5 common with CATI	8 Themes, 6 common with CATI and 4 common with FGDs
Open-Ended Question 2	Yes	9 Themes	10 Themes, 6 common with CATI	4 Themes, no unique themes
	No	9 Themes	10 Themes, 6 common with CATI	6 Themes, no unique themes
Open-Ended Question 3	-	11 Themes	10 Themes, 9 common with CATI	9 Themes, 1 unique theme
Open-Ended Question 4	-	12 Themes	13 Themes, 10 common with CATI	10 Themes, 1 unique theme
Open-Ended Question 5	Yes	8 Themes	—	8 Themes, 1 unique theme
	No	3 Themes	—	4 Themes, 2 unique themes

*Note*. CATI = computer-assisted telephone interviews; FGDs = focus group discussions; IR-SMS = interactive radio–short message service method.

Qualitative data from FGDs contained the most detail, while the CATI and interactive radio–SMS responses were often short without much elaboration. For example, all methods identified the weather as being an important reason why communities were/were not at risk, with cold weather reported to reduce cholera risk and hot weather increasing risk. In the CATI and interactive radio–SMS responses, reasons given were often very brief. However in FGDs, answers were expanded and linked hot weather with poor hygiene practices. Below are examples of the difference in responses:

Yes, the disease breaks out mostly in villages, during the hot season for example March and April and this causes bad hygiene, the community needs awareness on how to maintain hygiene and use chlorine where necessary. (FGD participant)

Yes, it affects people during the hot and rainy season and it is caused by lack of proper hygiene. (FGD participant)

Yes because of the hot weather. (Interactive radio–SMS participant)

Outbreak occurs a lot during the hot season but now we are not at risk. (CATI participant)

#### Levels of Agreement

We found that among participants in FGDs, unlike those in CATI and interactive radio–SMS, there was more consensus of views expressed in response to binary questions. This was particularly stark in FGDs with men and those living in internally displaced people’s camps. For example, in two male FGDs, all participants reported believing their communities were at risk of cholera and believing that their communities were not prepared for a cholera outbreak. Similar views were reported from the FGD with women living in internally displaced people’s camps. In the CATI or interactive radio–SMS samples, there were divergent views among the demographic groups.

### Quantitative and Integrated Results

#### Completeness

Extensive work has documented the significant reduction in completion rates associated with an increase in SMS-based survey length (Kongsgard et al., 2014). Low item response is therefore a possible threat to both the external and internal validity of interactive radio–SMS data. For this evaluation, interactive radio–SMS data from the original project were filtered to include individuals who reported residing in Banaadir and reported being at least 18 years (*N* = 385), excluding 6,303 individuals from these analyses^1^.

Completeness, defined as the proportion of messages in each data set (CATI and interactive radio–SMS) with a valid response, was calculated for only demographic and follow-up variables as the interactive radio–SMS method does not aim to have the same individuals participate in answering the open-ended questions across all shows. Table 5 provides a breakdown of the percentage complete for each variable in the two data sets. The CATI data set was more complete than the interactive radio–SMS data set.

**Table 5.** Summary of Completeness of Each Demographic and Follow-Up Variable Within the Interactive Radio–Short Message Service and Computer-Assisted Telephone Interview Data Sets.

Variable	Completeness in IR-SMS data set (%)	Completeness in CATI data set (%)
	(*N* = 385)	(*N* = 1,330)
District	—	100
Urban/rural	92.2	100
Gender	92.7	87.7
Age	—	87.7
Level of education	79.5	100
Living in IDP camp	89.4	100
Household sickness	73.5	100
Cholera vaccination	73.5	100

*Note*. Completeness for district and age are not presented as the original IR-SMS data set was restricted to those aged 18 years and older resident in Banaadir. IR-SMS = interactive radio–short message service method; CATI = computer-assisted telephone interviews; IDP = internally displaced people.

Interactive radio–SMS participation across open-ended questions varied week to week, with the following numbers of individuals responding to each question: 70, 31, 14, 27, and 128 (Questions 1-5, respectively)^2^; while all CATI participants responded to all the open-ended questions (*N* = 1,330).

#### Descriptive Demographic Comparisons

Sampling and coverage error are potential threats to the external validity of the interactive radio–SMS method. Biases arising from self-selection of interactive radio–SMS participants may result in some population groups being under/over represented and entire demographic groups omitted from samples. Relevant factors include the following: access to/use of media and technology; levels of literacy, trust in presenters and media houses; perception of interactive shows and others who participate; confidence to participate; language. Even when the entire range of demographic groups are reached, participating individuals are unlikely to be representative of their groups (gender, age, location) because they are not a random sample of those groups.

Table 6 summarizes the demographic characteristics of the two study populations. The CATI sample represents the general population and is the benchmark to which the interactive radio–SMS sample is evaluated. Comparing the demographic profiles of participants between the two methods, it is clear that the external validity of the interactive radio–SMS method is low, given the clear biases in the demographic composition of the interactive radio–SMS study population. Interactive radio–SMS participants were disproportionately male, urban residents, younger in age and more educated compared with the CATI sample. Interestingly, the interactive radio–SMS population included a larger proportion of internally displaced persons.

**Table 6.** Demographic Characteristics of Respondents in Interactive Radio–Short Message Service and Computer-Assisted Telephone Interview Data Sets.

Variable	CATI (%) [95% CI]	IR-SMS (%)
Gender (female)	58.7 [58.1, 59.3], *N* = 1166	44.5, *N* = 357
Age (years)		
18-19	7.0	33.8
20-24	28.6	35.8
25-29	24.5	15.6
30-34	13.3	6.8
35-39	9.6	2.1
40+	17.0	6.0
Younger than 25	35.6[35.2, 36.1], *N* = 1,167	69.6, *N* = 385
Education		
No schooling	21.1	5.6
Primary	10.8	12.1
Secondary	18.5	40.5
College/university	40.3	37.6
Islamic studies	9.2	3.6
Other	0.0	0.7
Secondary or more	58.8 [58.2, 59.4], *N* = 1,330	78.1, *N* = 306
Living in an urban area	85.7 [85.0, 86.4], *N* = 1330	93.2, *N* = 355
IDP	12.2 [11.9, 12.5], *N* = 1,330	18.0, *N* = 344

*Note*. CATI = computer-assisted telephone interviews; IR-SMS = interactive radio–short message service method; CI = confidence interval; IDP = internally displaced people.

#### Level-Oriented Comparisons

Comparisons of proportions of participants who answered yes to the binary open-ended and follow-up questions revealed that there was convergent validity for the Open-Ended Questions 1 and 5 and the follow-up question about cholera vaccination (Table 7). The differences in percentage points between the two methods ranged from 2.6 to 3.8 percentage points for Open-Ended Question 1 and 2.4-3.5 percentage points for Open-Ended Question 5. A very small sample of individuals responded to Open-Question 2 in the interactive radio–SMS data set and therefore comparisons are likely to be biased. The percentage of people who answered that they were vaccinated against cholera through interactive radio–SMS was within the boundaries of the confidence interval of the group that answered through CATI. For the follow-up question of whether there was anyone in their household who fell sick with cholera, the interactive radio–SMS responses were overestimated by between 9.9 and 10.4 percentage points, suggesting a possible bias due to individuals from households with cholera cases being more likely to participate in the radio shows.

**Table 7.** Percentage of Respondents Who Answered Yes to the Open-Ended and Follow-up Questions in Interactive Radio–Short Message Service and Computer-Assisted Telephone Interview Data Sets.

	CATI (%) [95% CI], *N* = 1330	IR-SMS, *N* (%)	Difference in percentage points between CATI and IR-SMS samples (min–max)
Open-Ended Question 1	70.3 [69.7, 70.9]	67.1%, *N*=70	2.6-3.8
Open-Ended Question 2	59.4 [58.8, 60.0]	77.4%, *N*=31	17.4-18.6
Open-Ended Question 5	95.8 [95.0, ,96.5]	89.1%, *N*=128	5.9-7.4
Cholera household	13.5 [13.3, 13.8]	23.7%, *N*=283	9.9-10.4
Cholera vaccination	29.8 [24.4, 30.3]	27.9%, *N*=283	2.4-3.5

*Note*. CATI = computer-assisted telephone interviews; IR-SMS = interactive radio–short message service method; CI = confidence interval.

#### Structure-Oriented Comparisons

Results from analyses assessing the association between views and demographic characteristics were limited in the interactive radio–SMS data set due to the small sample sizes with the exception of the follow-up question about cholera in the household (Table 8). This further supports the hypothesis of the self-selection of respondents in the interactive radio–SMS method based on cholera experience in their household. Within the CATI data set there were a number of associations that remained statistically significant even after adjusting for all other demographic variables. For example in Open-Ended Question 1, men were more likely to respond that they believed their communities were at risk of cholera; while in Open-Ended Question 2, women, respondents in internally displaced people’s camps, and those aged ≥25 years were more likely to respond that they believed their communities were prepared for a cholera outbreak (Table 8).

**Table 8.** Odds Ratios Between Binary Responses to Open-Ended Questions and Demographic Characteristics for Computer-Assisted Telephone Interview and Interactive Radio–Short Message Service Participants Adjusted for all Other Demographic Variables.

	Gender (ref = female)	Rural/urban (ref = urban)	IDP (ref = non-IDP)	Age group (ref ≤ 25 years)
CATI (*N* = 1,166)				
Open-Ended Question 1	**1.41 [1.08, 1.84]**	0.73 [0.52, 1.03]	1.08 [0.74, 1.59]	1.25 [0.96, 1.62]
Open-Ended Question 2	**0.49 [0.38, 0.62]**	1.26 [0.89, 1.79]	**2.19 [1.41, 3.41]**	**1.43 [1.11, 1.84]**
Open-Ended Question 5	0.73 [0.41, 1.29]	0.54 [0.27, 1.03]	0.90 [0.40, 2.02]	**0.51 [0.26, 0.99]**
Cholera household	0.90 [0.63, 1.29]	1.22 [0.79, 1.90]	**2.64 [1.75, 4.01]**	**1.59 [1.08, 2.34]**
Cholera vaccination	**0.65 [0.50, 0.85]**	**1.56 [1.11, 2.18]**	1.18 [0.81, 1.71]	1.00 [0.77, 1.31]
IR-SMS (*N*)				
Open-Ended Question 1 (*N*=43)	1.30 [0.37, 4.59]	—	0.70 [0.14, 3.42]	1.97 [0.33, 11.80]
Open-Ended question 2 (*N*=21)	2.16 [0.22, 20.90]	0.58 [0.02, 17.11]	—	1.56 [0.19, 12.70]
Open-ended question 5 (*N*=116)	0.48 [0.11, 2.08]	0.35 [0.03, 4.14]	1.01 [0.17, 6.19]	0.62 [0.14, 2.75]
Cholera household (*N*=222)	0.84 [0.43, 1.63]	**3.24 [1.03, 10.18]**	**2.59 [1.15, 5.79]**	**2.55 [1.28, 5.08]**
Cholera vaccination (*N*=224)	1.36 [0.75, 2.47]	0.86 [0.26, 2.87]	1.26 [0.58, 2.73]	0.79 [0.41, 1.51]

*Note*. Statistically significant figures are in bold. CATI = computer-assisted telephone interviews; IR-SMS = interactive radio–short message service method; ref = reference group; IDP = internally displaced people; CI = confidence interval.

#### Regressions

In regression analyses, the effect of mode on the binary responses to open-ended questions was not statistically significant (*p* > .05), even after controlling for demographic variables (Table 9). This suggests that disparities in results between CATI and interactive radio–SMS are due to differences in the composition of participants, which is a result of the nonprobabilistic sampling in the interactive radio–SMS method. However, for the follow-up question about cholera in the household, we found that mode affected the binary responses. This result further reiterates the bias of the interactive radio–SMS sample toward individuals who are interested-in/affected-by the topic, suggesting that motivation to participate does play an important role in respondent’s self-selection in the interactive radio–SMS method. This self-interest bias could be beneficial in certain health or humanitarian emergencies as people who are affected may be more easily reached through interactive radio–SMS.

**Table 9.** Odds Ratios Between Binary Responses to Open-Ended Questions and Mode (Computer-Assisted Telephone Interview vs. Interactive Radio–Short Message Service) Adjusted for All Other Demographic Variables.

Variable (*N*)	Estimate (ref = IR-SMS)	95% CI	*p*
Open-Ended question 1 (*N*=43)	−0.29	[−0.66, 0.43]	.51
Open-Ended question 2 (*N*=21)	−0.29	[−0.48, 0.59]	.63
Open-Ended question 5 (*N*=116)	−0.73	[−1.53, 0.47]	.12
Cholera household (*N*=222)	**0.87**	**[0.21, 4.09]**	**<.001**
Cholera vaccination (*N*=224)	0.13	[0.18, 0.71]	.47

*Note*. Statistically significant figures are in bold. IR-SMS = interactive radio–short message service method; ref = reference group; 95% CI = 95 percentage of confidence interval.

## Discussion

Interactive radio–SMS is a mixed methods approach that offers opportunities to enhance social research in LMICs in the context of the COVID-19 pandemic, so long as its limitations are fully appreciated. Below, we discuss these methodological strengths and limitations; highlight key features of the method that warrant consideration in order to minimize limitations and maximize strengths; outline specific use-cases for the interactive radio–SMS method in public health and other fields; and finally discuss the contribution of this study to the field of mixed methods research.

First, our evaluation found that threats to the credibility of the qualitative component of the interactive radio–SMS method were low as major themes were found across all three methods (convergence). Thus, interactive radio–SMS can shed light on potentially important local sociocultural beliefs and social norms, such as levels of rumor, denial, or negative stigma, including among more vulnerable populations such as internally displaced persons. Furthermore, and importantly in the COVID-19 pandemic, interactive radio–SMS can overcome access and movement restrictions on face-to-face research while offering an alternative broadcast media base for capturing social discussions that also distinguishes it from individual phone based surveys.

In Somalia, for example, the interactive radio–SMS method was used as a rapid diagnostic tool in the early days of the COVID-19 pandemic to understand perspectives of Somali respondents in south-central Somalia and Puntland on the outbreak (Africa’s Voices Foundation, 2020). These results found that among individuals who replied, there was a large proportion who interpreted this new threat through a religious hope/practice frame. Furthermore, when qualitative themes were transformed into quantitative measures and analyzed alongside demographic data, among respondents from Banaadir, those who were internally displaced persons were significantly more likely to convey thoughts on COVID-19 that involved rumor, stigma, or misinformation within a religious frame than those from host communities. These findings highlighted the need for further investigation into how religious leaders could be likely trusted sources of advice and the spread of rumors and misinformation, particularly in internally displaced persons communities.

On the other hand, the nature of the interactive radio–SMS method carries inherent limitations which affect the interpretation and conclusions drawn from integrated results. In line with previous research (Lopes and Srinivasan, 2014), our study identified threats to the validity legitimation of the quantitative and integrated components of the interactive radio–SMS method linked to biases associated with self-selection, low response rates, and incompleteness of data. Considering that the level of analysis of interactive radio–SMS studies is ideological, comprising belief systems, social representations, and social norms of societies (Doise & Valentim, 2015; World Bank Group, 2015), the lack of representativeness is not as important as in opinion surveys or epidemiological studies whose level of analysis is the individual (Rothman et al., 2013). The knowledge about the community beliefs can be applied beyond the specific group of participants in the radio shows to the community they belong to. However, the findings cannot be generalized to groups not considered in the study (e.g., nonparticipants, digitally excluded) or to other units of analysis such as individuals or countries, to avoid ecological and atomistic fallacies (Subramanian et al., 2009).

Future work using the interactive radio–SMS method should therefore be careful with the presentation and interpretation of integrated results, focusing on hypothesis generation, to avoid misrepresentation and inaccuracies which might arise due to generalizing results to nonparticipating groups. This is particularly important when using findings to inform COVID-19 or other health emergency response plans. Based on the results from our evaluation we recommend using the interactive radio–SMS method principally for hypothesis generation or to identify critical research questions which then need to be further explored. Given its limitations, researchers should be very cautious to use the method in isolation to inform programmatic interventions. Instead following methodological best practices in social sciences, triangulation of results from interactive radio–SMS through cross-validation with other methods should be sought.

It is important to note that the threats to validity we identified in this study will vary in magnitude across different projects using the interactive radio–SMS method, with some being more prone to certain threats than others (e.g., social desirability). Future projects involving the interactive radio–SMS method should endeavor to understand how these threats compromise the results and find ways to minimize them through the project design. Design options include, for example, using hypothetical scenarios to circumvent social desirability or avoiding controversial questions that may polarize community views. Key procedures that future research should consider to minimize limitations associated with the method include: pretesting framing of open-ended questions (WHO, 2018); minimizing the number of SMS questions asked; using strategies to promote more inclusive and higher participation (Internews, 2015); automating routines to deal with processing and coding errors; and feeding back results to audiences in an appropriate format to further validate findings. Furthermore, ethical questions around participation and consent of minors as well as handling and responding to sensitive information shared through the SMS interactions should also be carefully considered.

With this understanding, there are a number of research problems in social sciences in LMICs, including public health emergencies such as COVID-19 but also well beyond (education, natural disasters, livelihoods, gender equity, clean water and sanitation, conservation, governance), where interactive radio–SMS as a mixed methods approach might be particularly beneficial. These include for the following: (1) inspection of predominant and/or unexpected community views or beliefs, and if inclusion and participation are high of less frequent community beliefs which can then be further explored; (2) continued engagement with communities through iterative and evolving discussions for monitoring risk, trust and behavioral or social change; (3) deeper understanding of social and physical barriers for implementation of interventions among certain subgroups; (4) refining and focusing open-ended questions based on answers to previous questions (e.g., to better understand or address community fears or misconceptions); (5) potentially reaching digitally connected populations with radio coverage in insecure contexts or geographically hard to reach areas.

## Contribution to the Field of Mixed Methods

This article contributes to the field of mixed methods in several ways. First, we present the application of a mixed methods approach which addresses a specific challenge raised by COVID-19, namely utilizing media and communications technologies for social research, particularly in LMICs (WHO, 2020). In doing so, we detail an applied mixed methods approach that integrates qualitative and quantitative concepts, methods, and data providing a reference for future studies which want to use interactive radio–SMS for social research responding to public health crises.

Second, interactive radio–SMS as a mixed methods approach combines the depth of qualitative analyses with the breadth of quantitative research in a noncompartmentalized way, contributing to expanding the range of mixed methods designs (Maxwell, 2016). The transformation of primary qualitative data and subsequent integrated analyses in our approach utilities both types of data to answer related aspects of the same research question thereby further enriching findings.

Finally, our evaluation of the interactive radio–SMS method provides evidence of how some validity threats influence the results of mixed methods. Determining when and in what circumstances the benefits of a mixed design outweigh the limitations associated with integration is crucial to effectively applying the method and correctly interpret findings. This is especially important for newer methods which have not been formally evaluated (e.g., interactive radio–SMS). Our article therefore contributes to the evidence base on the method, detailing the impact of validity threats on findings and informing judgements about when to use the method and what conclusions can be drawn from findings given its limitations.

## Conclusion

Interactive radio–SMS represents a mixed methods approach of particular relevance in LMICs and during emergencies such as COVID-19. By utilizing media and digital technologies and accommodating local languages, this method holds promise for contributing research findings on sociocultural phenomena crucial to effective response planning. However, given its limitations there is a need for high levels of care in research design, implementation, presentation, and interpretation of results. Future research of the interactive radio–SMS method should build on this evaluation to investigate how different topics affect the quality of answers and also how the radio shows change or crystallize the views of participants and audiences, an area that remains unexplored. Overall, with the global prioritization of COVID-19, this research provides a timely illustration of potentially new applications of a mixed methods approach for social research in public health emergencies.

## Notes

1. Individuals were excluded for the following reasons: 1,144 individuals were either resident outside of Banaadir or aged younger than 18 years; 450 individuals did not report a district of resident; 2,063 individuals did not report their age; 2,646 did not report a district of residence nor their age.

2. The total number of individual responses received in the original interactive radio–SMS data set were 3,315; 1,201; 748; 1,111; and 2,458 for Open-Questions 1 to 5, respectively.
